# Glioblastoma Surgery Imaging–Reporting and Data System: Validation and Performance of the Automated Segmentation Task

**DOI:** 10.3390/cancers13184674

**Published:** 2021-09-17

**Authors:** David Bouget, Roelant S. Eijgelaar, André Pedersen, Ivar Kommers, Hilko Ardon, Frederik Barkhof, Lorenzo Bello, Mitchel S. Berger, Marco Conti Nibali, Julia Furtner, Even Hovig Fyllingen, Shawn Hervey-Jumper, Albert J. S. Idema, Barbara Kiesel, Alfred Kloet, Emmanuel Mandonnet, Domenique M. J. Müller, Pierre A. Robe, Marco Rossi, Lisa M. Sagberg, Tommaso Sciortino, Wimar A. Van den Brink, Michiel Wagemakers, Georg Widhalm, Marnix G. Witte, Aeilko H. Zwinderman, Ingerid Reinertsen, Philip C. De Witt Hamer, Ole Solheim

**Affiliations:** 1 Department of Health Research, SINTEF Digital, NO-7465 Trondheim, Norway; andre.pedersen@sintef.no (A.P.); ingerid.reinertsen@sintef.no (I.R.); 2 Department of Neurosurgery, Amsterdam University Medical Centers, Vrije Universiteit, 1081 HV Amsterdam, The Netherlands; r.eijgelaar@amsterdamumc.nl (R.S.E.); i.kommers@amsterdamumc.nl (I.K.); dmj.muller@amsterdamumc.nl (D.M.J.M.); p.dewitthamer@amsterdamumc.nl (P.C.D.W.H.); 3 Cancer Center Amsterdam, Brain Tumor Center, Amsterdam University Medical Centers, 1081 HV Amsterdam, The Netherlands; 4 Department of Neurosurgery, Twee Steden Hospital, 5042 AD Tilburg, The Netherlands; h.ardon@etz.nl; 5 Department of Radiology and Nuclear Medicine, Amsterdam University Medical Centers, Vrije Universiteit, 1081 HV Amsterdam, The Netherlands; f.barkhof@amsterdamumc.nl; 6 Institutes of Neurology and Healthcare Engineering, University College London, London WC1E 6BT, UK; 7 Neurosurgical Oncology Unit, Department of Oncology and Hemato-Oncology, Humanitas Research Hospital, Università Degli Studi di Milano, 20122 Milano, Italy; lorenzo.bello@unimi.it (L.B.); marco.conti@unimi.it (M.C.N.); marco.rossi2@unimi.it (M.R.); tommaso.sciortino@unimi.it (T.S.); 8 Department of Neurological Surgery, University of California, San Francisco, CA 94143, USA; mitchel.berger@ucsf.edu (M.S.B.); shawn.hervey-jumper@ucsf.edu (S.H.-J.); 9 Department of Biomedical Imaging and Image-Guided Therapy, Medical University Vienna, 1090 Wien, Austria; Julia.Furtner@meduniwien.ac.at; 10 Department of Circulation and Medical Imaging, Norwegian University of Science and Technology, NO-7491 Trondheim, Norway; even.h.fyllingen@ntnu.no; 11 Department of Radiology and Nuclear Medicine, St. Olavs Hospital, Trondheim University Hospital, NO-7030 Trondheim, Norway; 12 Department of Neurosurgery, Northwest Clinics, 1815 JD Alkmaar, The Netherlands; A.J.S.Idema@nwz.nl; 13 Department of Neurosurgery, Medical University Vienna, 1090 Wien, Austria; barbara.kiesel@meduniwien.ac.at (B.K.); georg.widhalm@meduniwien.ac.at (G.W.); 14 Department of Neurosurgery, Haaglanden Medical Center, 2512 VA The Hague, The Netherlands; a.kloet@mchaaglanden.nl; 15 Department of Neurological Surgery, Hôpital Lariboisière, 75010 Paris, France; emmanuel.mandonnet@aphp.fr; 16 Department of Neurology and Neurosurgery, University Medical Center Utrecht, 3584 CX Utrecht, The Netherlands; P.Robe@umcutrecht.nl; 17 Department of Neurosurgery, St. Olavs Hospital, Trondheim University Hospital, NO-7030 Trondheim, Norway; lisa.millgard.sagberg@ntnu.no; 18 Department of Neurosurgery, Isala Hospital Zwolle, 8025 AB Zwolle, The Netherlands; brink@neurochirurgie-zwolle.nl; 19 Department of Neurosurgery, University Medical Center Groningen, University of Groningen, 9713 GZ Groningen, The Netherlands; m.wagemakers@umcg.nl; 20 Department of Radiation Oncology, The Netherlands Cancer Institute, 1066 CX Amsterdam, The Netherlands; m.witte@nki.nl; 21 Department of Clinical Epidemiology and Biostatistics, Amsterdam University Medical Centers, 1105 AZ Amsterdam, The Netherlands; a.h.zwinderman@amsterdamumc.nl (A.H.Z.); ole.solheim@ntnu.no (O.S.); 22 Department of Neuromedicine and Movement Science, Norwegian University of Science and Technology, NO-7491 Trondheim, Norway

**Keywords:** glioblastoma, deep learning, 3D segmentation, computer-assisted image processing, magnetic resonance imaging, neuroimaging

## Abstract

**Simple Summary:**

Neurosurgical decisions for patients with glioblastoma depend on visual inspection of a preoperative MR scan to determine the tumor characteristics. To avoid subjective estimates and manual tumor delineation, automatic methods and standard reporting are necessary. We compared and extensively assessed the performances of two deep learning architectures on the task of automatic tumor segmentation. A total of 1887 patients from 14 institutions, manually delineated by a human rater, were compared to automated segmentations generated by neural networks. The automated segmentations were in excellent agreement with the manual segmentations, and external validity, as well as generalizability were demonstrated. Together with automatic tumor feature computation and standardized reporting, our Glioblastoma Surgery Imaging Reporting And Data System (GSI-RADS) exhibited the potential for more accurate data-driven clinical decisions. The trained models and software are open-source and open-access, enabling comparisons among surgical cohorts, multicenter trials, and patient registries.

**Abstract:**

For patients with presumed glioblastoma, essential tumor characteristics are determined from preoperative MR images to optimize the treatment strategy. This procedure is time-consuming and subjective, if performed by crude eyeballing or manually. The standardized GSI-RADS aims to provide neurosurgeons with automatic tumor segmentations to extract tumor features rapidly and objectively. In this study, we improved automatic tumor segmentation and compared the agreement with manual raters, describe the technical details of the different components of GSI-RADS, and determined their speed. Two recent neural network architectures were considered for the segmentation task: nnU-Net and AGU-Net. Two preprocessing schemes were introduced to investigate the tradeoff between performance and processing speed. A summarized description of the tumor feature extraction and standardized reporting process is included. The trained architectures for automatic segmentation and the code for computing the standardized report are distributed as open-source and as open-access software. Validation studies were performed on a dataset of 1594 gadolinium-enhanced T1-weighted MRI volumes from 13 hospitals and 293 T1-weighted MRI volumes from the BraTS challenge. The glioblastoma tumor core segmentation reached a Dice score slightly below 90%, a patientwise F1-score close to 99%, and a 95th percentile Hausdorff distance slightly below 4.0 mm on average with either architecture and the heavy preprocessing scheme. A patient MRI volume can be segmented in less than one minute, and a standardized report can be generated in up to five minutes. The proposed GSI-RADS software showed robust performance on a large collection of MRI volumes from various hospitals and generated results within a reasonable runtime.

## 1. Introduction

Gliomas represent the primary central nervous system pathology with the highest incidence rate in adults [1]. Occurring from the glial tissue, gliomas can be classified into subtypes based on histology and are graded from I to IV following the morphology and malignant behavior as specified in the World Health Organization (WHO) classification [2]. Stage IV glioma, also known as glioblastoma (GBM), is the most invasive brain tumor with highly diffuse infiltrative characteristics, making it extremely lethal with a median survival of 12 months when treated by radiotherapy [3]. These tumors are very heterogeneous (i.e., edema, enhancing, and nonenhancing core) and are depicted by different intensity profiles across the various Magnetic Resonance Imaging (MRI) sequences (i.e., T1-weighted, T2, or FLAIR (FLuid Attenuated Inversion Recovery)). Heterogeneity in glioblastoma does not only complicate the diagnostic task, but also the prognosis and survival prediction using MR imaging [4]. The quantitative analysis of MRI volumes to extract imaging features through advanced computational algorithms can lead to important and reproducible evidence finding [5].

However, glioblastoma segmentation on MRI is generally not part of standard care for multiple reasons. For the most part, manual segmentation or assessment by radiologists is time-consuming and subject to intra- and inter-rater variations that are difficult to characterize [6], and therefore, this is rarely done in clinical routine. Informative tumor features such as location or volume are often estimated from the images solely based on eyeballing or crude measures of tumor diameters [7]. The range of applications for GBM segmentation is not solely limited to surgical or radiotherapeutic planning. The extent of resection [8], volume growth [9], quantification of the effects of interventions [10], and probability maps of tumors and resections [11] can all be derived from accurate segmentation. These applications demand a high level of robustness, repeatability, and ability to generalize. Advances in the field of deep learning and artificial intelligence have shown that automatic segmentation can perform as well as expert radiologists on various tasks [12,13]. Well-established healthcare manufacturers (e.g., Siemens, GE) have started providing digital health solutions including Artificial Intelligence (AI) companions integrating multimodality imaging decision support. The AI-Rad Companion from Siemens, designed to reduce the burden of basic repetitive tasks (e.g., segmentation) and to increase diagnostic precision, already presents a module for brain MRI processing (https://grand-challenge.org/aiforradiology/product/siemens-rad-companion-brain-mr/, accessed on 16 September 2021).

Patient-specific planning and follow-up could be enabled by automatic and robust characterization of the tumor following segmentation. The automatic generation of standardized reports is bound to the same principles and requirements as the segmentation task, where robustness and repeatability are key. Standard Reporting And Data Systems (RADSs) have been proposed for several solid tumors, such as in prostate cancer [14], breast cancer [15], head and neck cancer [16], and lung cancer [17]. Common rules for imaging techniques, reports’ terminology, and definitions of tumor features and treatment response were defined. Image-derived quantitative measurements, also known as radiomics [18], were an attempt to characterize tumor phenotypes for use in computer-aided diagnosis tools [19]. Radiomics can contribute to RADSs, but are limited in their clinical relevance. Tumor shape and morphology hold a descriptive and informative power and can be provided directly to the clinician. On the other hand, measurements such as intensity histograms or tumor texture are impossible to interpret in their raw form. A tremendous contribution from such systems is hence the automatic result generation that is not prone to rater variability, whether due to limited available time, stress, tiredness level, or level of expertise. In order to alleviate clinicians’ burden and generate trustworthy medical reports, the need for automatic tumor segmentation, together with the computation of standardized measurements, has become apparent.

In the field of brain tumor segmentation, the majority of studies have focused on gliomas under the impulsion of the BraTS challenge and its publicly available dataset [20,21]. The latest iteration of the dataset from 2020 contains 494 patients with a combination of HGGs (High-Grade Gliomas) and LGGs (Low-Grade Gliomas), with four MRI sequences available each (i.e., T1, T1Gd, T2, FLAIR). All data were delineated manually, with a differentiation among Gd-enhancing tumor, peritumoral edema, and the necrotic and nonenhancing tumor core. Over the last five years, tremendous progress has been made on segmentation from advances in artificial intelligence and deep learning. Initial works were performed in 2D due to the lack of hardware resources, and each axial image (slice) from a 3D MRI volume was to be processed sequentially [22]. In order to benefit from more global information, later approaches evolved towards slabwise/patchwise strategies over 3D neural network architectures [23]. Alternatively, approaches leveraging a downsampled version of the full 3D MRI volume or ensemble designs combining both strategies were also suggested to further boost segmentation performance [24]. As part of the best-performing approaches for glioma segmentation, over the BraTS challenge dataset, we can find nnU-Net [25] and H^2^NF-Net [26]. The former uses a 3D U-Net backbone architecture and operates in a 3D patchwise fashion, determining the best preprocessing steps from a careful analysis of the input dataset characteristics. If deemed necessary, a cascade design is enabled whereby the first model is trained on the downsampled inputs before training the patchwise model. The latter is a hybrid high-resolution and nonlocal feature network, also able to train both single or cascaded models. A parallel multiscale fusion module provides the foundation to maintain strong feature representation and aggregate multiscale contextual information. Overall, segmentation performance around an 80% Dice score for the contrast-enhancing tumor core was reached. Regarding other brain tumor types, such as meningioma, the segmentation topic has been less investigated with a few studies performed using in-house datasets and less advanced architectures [6,27,28,29]. In a recent work, competitive segmentation and detection performances were achieved with the AGU-Net architecture [30]. A combination of deep supervision, multiscale input, and attention modules built around a 3D U-Net backbone enabled the network to efficiently leverage the global context while retaining local information. Independent of the application, existing architectures present a high potential for transfer.

Standardized RADSs for brain tumors have been sparsely investigated. A RADS for post-treatment brain tumors was recently proposed [31]. A scoring system guiding the neuroradiologist through the interpretation of the post-treatment MR image was mainly provided, using a structured set of rules. However, neither automatic tumor segmentation, nor image analysis were included. As such, the system is another set of criteria evaluated by the neuroradiologist and does not resolve the fundamental challenge of the objective and accurate characterization of brain tumors in MRI. In our previous study, the clinical utility of the proposed GSI-RADS for standardized glioblastoma reporting was studied [32]. The performance of automatic segmentation was compared to manual segmentation as the basis for the computation of relevant tumor features. The tumor features computed based on automatic segmentation were in excellent agreement with the features computed based on manual segmentation. In this paper, we further extend and complement this work by improving the initial automatic segmentation model included in GSI-RADS, support for better clinical measurements. In addition, a thorough comparison between two state-of-the-art neural network architectures in terms of segmentation and detection performances is performed. In addition, a summarized description of the proposed tumor feature extraction process, as well as the GSI-RADS software is provided.

In our previous study, the need and potential advantages in clinical practice for automated segmentation and standardized reporting were addressed [32]. In this study, the focus is brought towards tumor core segmentation (i.e., contrast-enhancing tumor and necrotic tumor core) in gadolinium-enhanced T1-weighted MRI volumes for tumor feature computation and standard reporting for high-grade gliomas. The contributions are: (i) the training of robust segmentation models using two state-of-the-art convolutional neural network architectures and different data preprocessing schemes, (ii) extensive validation studies both across hospitals and using the BraTS challenge dataset as external benchmark, and (iii) open-access models, the inference code, and a stand-alone software.

## 2. Data

**Figure 1:**
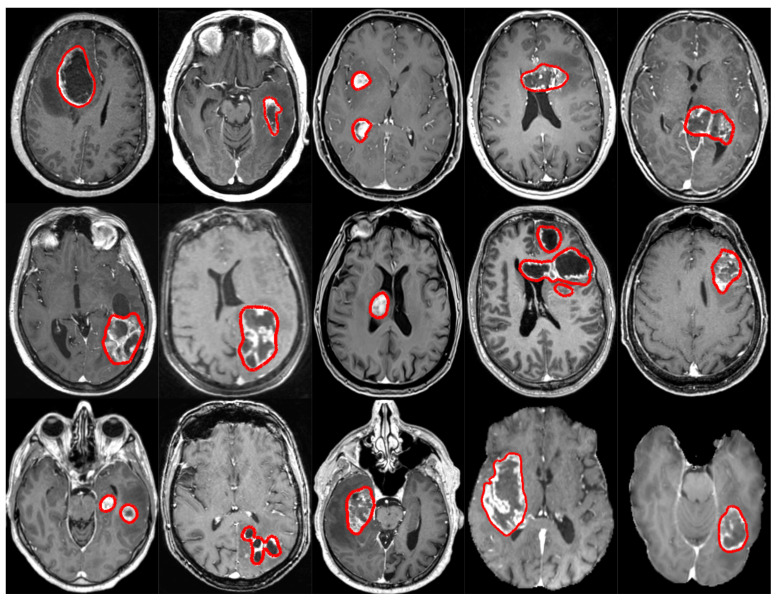
Example axial views of T1-weighted MRI volumes used in the study, with manual segmentations outlined in red. One case from each hospital is shown, and the rightmost two cases featured in the last row are from the BraTS challenge dataset.

A dataset comprised of a total of 1887 T1-weighted MRI volumes was put together, which has been further split into two subsets. The first subset, called GS1, contains 1594 unique T1-weighted MRI volumes collected from 13 hospitals [32]. The second subset, called GS2, corresponds to the 2020 edition of the BraTS challenge dataset [20]. Some examples from both subsets, with the manual segmentations outlined in red, are provided in Figure 1.

### 2.1. Subset 1 (GS1)

T1-weighted MRI volumes were collected from a total of 1594 patients from 13 different hospitals worldwide. The hospitals and number of patients from each were distributed as follows: 38 patients from the Northwest Clinics, Alkmaar, Netherlands (*ALK*); 97 patients from the Amsterdam University Medical Centers, location VU medical center, Netherlands (*AMS*); 86 patients from the University Medical Center Groningen, Netherlands (*GRO*); 103 patients from the Medical Center Haaglanden, the Hague, Netherlands (*HAG*); 75 patients from the Humanitas Research Hospital, Milano, Italy (*MIL*); 74 patients from the Hôpital Lariboisière, Paris, France (*PAR*); 134 patients from the University of California San Francisco Medical Center, U.S. (*SFR*); 49 patients from the Medical Center Slotervaart, Amsterdam, Netherlands (*SLO*); 153 patients from the St Elisabeth Hospital, Tilburg, Netherlands (*TIL*); 171 patients from the University Medical Center Utrecht, Netherlands (*UTR*); 83 patients from the Medical University Vienna, Austria (*VIE*); 72 patients from the Isala hospital, Zwolle, Netherlands (*ZWO*); 459 patients from the St. Olavs hospital, Trondheim University Hospital, Norway (STO). An in-depth description of the different cohorts can be found in a recent study [32].

All tumors were manually segmented in 3D using the assistance of external tools, such as a region-growing algorithm [33] or a grow-cut algorithm [34], followed by manual adjustment. The different trained annotators were supervised by neuroradiologists and neurosurgeons. In general, a tumor was defined as gadolinium-enhancing tissue on T1-weighted scans, including nonenhancing enclosed necrosis or cysts.

### 2.2. Subset 2 (GS2)

In the BraTS challenge dataset edition from 2020, a total of 293 patients with glioblastoma are featured in the training set. Only the T1-weighted MRI volumes and corresponding annotations were kept to constitute our second subset. Unfortunately, it is important to note that the original raw MRI volumes were not provided by the BraTS challenge, but rather volumes having undergone a series of preprocessing transformations such as brain masking (cf. the rightmost examples in the last row of Figure 1).

## 3. Methods

First, the pixelwise segmentation process, including data preprocessing, architectures, and training design, is introduced in Section 3.1. Second, using the generated automatic segmentation as the input, the tumor feature extraction process is summarized in Section 3.2. Finally, an overview of the developed software for using the proposed methods alongside the standardized reporting strategy is given in Section 3.3.

### 3.1. Segmentation

In order to produce competitive pixelwise segmentation models, the following convolutional neural network architectures were selected: nnU-Net [25] and AGU-Net [30]. The nnU-Net framework obtained the best results on high-grade glioma segmentation over the BraTS challenge dataset. Overall, the framework was built to provide a standardized baseline for medical image segmentation, using a 3D U-Net architecture with residual connections automatically optimized for the specific input dataset. The latter architecture has been shown to be competitive and able to generalize well in the task of meningioma segmentation, over a dataset of 600 patients. Solely leveraging T1-weighed MRI volumes as the input, the architecture is made of only one stage directly leveraging the whole 3D MRI volume at a lower resolution than the original. Additional schemes are used to preserve the details (i.e., multiscale input and deep supervision), and attention mechanisms are further exploited to fine-tune the features towards the specificity of the segmentation target.

In previous studies on pixelwise semantic segmentation, various preprocessing steps were investigated with a consensus around intensity normalization and resizing or rescaling operations as the most common steps [35]. In the specific field of medical image segmentation, and more so for MRI volumes, an additional array of operations can be used from bias correction to skull stripping [36,37]. From the amount of steps and the complexity of the preprocessing strategy, a tradeoff between segmentation performance and speed to segment one MRI volume is to be found. In this study, two different preprocessing schemes were investigated with an increasing degree of complexity and required computing time (P1 and P2), described further for each architecture. For reference, the models used for assessing the quality of the automatic tumor feature extraction in our previous study [32] were trained using the AGU-Net architecture under the P1 preprocessing scheme.

#### 3.1.1. Specifications for nnU-Net

Since the nnU-Net framework was used, most of the following parameters were automatically inferred from the characteristics of the dataset:

##### Preprocessing

1. Light (P1): (i) cropping tightly around the patient’s head, (ii) z-score intensity normalization (zero mean, unit variance), and (iii) resampling to a spacing of $0.98\times 0.99\times 1.0\;{\mathrm{mm}}^{3}$ using spline interpolation of order 3;
2. Heavy (P2): (i) brain segmentation and brain-masking, (ii) cropping tightly around the patient’s brain, (iii) z-score intensity normalization (zero mean, unit variance), and (iv) resampling to a spacing of $0.98\times 0.99\times 1.0\;{\mathrm{mm}}^{3}$ using spline interpolation of order 3.

The brain segmentation and masking step was performed using the HD-BET approach [38]. Every pixel of the original MRI volume not belonging to the brain class had its intensity set to zero. The median volume dimension of (cropped) volumes was 261×220×180 voxels for P1 and 169×141×140 voxels for P2, both with a voxel spacing of $0.98\times 0.99\times 1.0\;{\mathrm{mm}}^{3}$.

##### Architecture Design

From the framework analysis of our dataset, the suggested best-performing architecture was the 3D full-resolution U-Net. This architecture was used for all experiments without using a cross-validation-based ensemble. The network used five levels, downsampling using strided convolution layers, and upsampling using transposed convolution layers. Kernel sizes of 3×3×3 voxels for all five levels and filter sizes of [32,64,128,256,320] were used. The loss function was a combination of the Dice score and cross-entropy. The stride values for the convolution layers were as follows, based on the preprocessing performed: strides for P1: [2,2,1],[2,2,2],[2,2,2],[2,2,2],[2,2,2], strides for P2: [2,2,2],[2,2,2],[2,2,2],[2,2,2],[2,2,2]. The input patch sizes were 160×128×112 voxels for P1 and 128×128×128 voxels for P2.

##### Network Training

All models were trained from scratch using the stochastic gradient descent with Nesterov momentum optimizer (momentum = 0.99) with a 0.01 initial learning rate, for 1000 epochs. One epoch was defined as iterations over 250 mini-batches of batch size 2. The learning rate was decayed throughout training following the “poly” learning rate policy with a 0.9 hyperparameter [39].

#### 3.1.2. Specifications for AGU-Net

##### Preprocessing

1. Light (P1): (i) resampling to an isotropic spacing of $1\;{\mathrm{mm}}^{3}$ using spline interpolation of order 1 from NiBabel (https://github.com/nipy/nibabel, accessed on 16 September 2021), (ii) cropping tightly around the patient’s head, (iii) volume resizing to match the architecture’s input size, and (iv) normalizing intensities to the range [0,1];
2. Heavy (P2): (i) resampling to an isotropic spacing of $1\;{\mathrm{mm}}^{3}$ using spline interpolation of order 1, (ii) brain segmentation and brain-masking, (iii) volume resizing to match the architecture’s input size, and (iv) zero-mean normalization of intensities.

The brain segmentation and masking step was performed using a modified 3D U-Net architecture trained on 100 T1-weighted MRI volumes with slabs of size 256×192×32 voxels. Every pixel of the original MRI volume not belonging to the brain class had its intensity set to zero, and the tightest bounding volume encompassing the non-null intensity value was kept.

##### Architecture Design

The final architecture was as described in the original article [30] with five levels and [16,32,128,256,256] as the filter sizes, with the input size set to 128×128×144 voxels. All modules including the multiscale input, deep supervision, and singe attention modules were enabled. The loss function was the class-averaged Dice loss, excluding the background class.

##### Network Training

All models were trained from scratch using the Adam optimizer with a $1\times 10^{-3}$ initial learning rate, stopped after 30 consecutive epochs without validation loss improvement. Given the large memory footprint, a batch size of 2 was used, further brought up to 32 using the concept of accumulated gradients. A typical data augmentation approach was used whereby the following transforms were applied on each training sample with a 50% probability: horizontal and vertical flipping, random rotation in the range $\left[-20^{\circ },20^{\circ }\right]$, and translation up to 10% of the axis dimension.

### 3.2. Clinical Feature Computation

In order to report clinical features in a standardized and reproducible fashion, the feature computation was performed after alignment to a standard reference space. In this study, the reference space was constituted by the symmetric Montreal Neurological Institute ICBM2009a atlas (MNI) [40,41].

The alignment process for a patient’s MRI volume consisted of two steps. First, a segmentation of the brain was performed, using the same model as described in Section 3.1.2. Then, the image-to-image registration was executed using the SyN diffeomorphic approach from ANTs [42]. As a preliminary step to maximize registration accuracy, the brain-masked versions of the MRI volumes were used, whereby all pixels not belonging to the brain had their intensities set to zero. The registration produced a set of transforms in order to perform the mapping between the patient space and the MNI space. The tumor segmentation, automatically generated from one of the trained models, was then registered to the reference space using the set of transforms. As a result of this procedure, the original MRI volume and automatically inferred tumor segmentation were transformed to the MNI space. Depending on the quality of the pixelwise segmentation approach used, a refinement step had to be included before computing the features in order to remove lingering noise. To do so, a binary closing morphological operator was applied with two iterations, using a spherical kernel with radius 2. Any object smaller than 0.1 mL was discarded, after employing a connected component approach.

From the refined and registered tumor segmentation mask, the following features were computed: laterality, volume, multifocality, resectability [43], location profile of cortical structures [44,45,46,47,48,49,50], and location profile of subcortical structures [51]. From the 233 extracted features, all were expressed in the MNI space with the exception of the tumor volume, expressed in the original patient space.

A detailed description of the tumor feature computation is provided in the following paragraphs, as a complement to the description in our previous study [32].

#### 3.2.1. Volume (2 Parameters)

This was computed as the product between the MRI volume slice spacing values (mm${}^{3}$) and the total amount of tumor voxels, and expressed in milliliters (mL) both in the patient and MNI space.

#### 3.2.2. Laterality (3 Parameters)

A laterality mask was created, splitting the MNI atlas in half. The percentage of the total tumor volume overlapping with each hemisphere was computed and reported. In addition, a midline crossing Boolean value is provided if the tumor was overlapping both hemispheres by at least 1 voxel.

#### 3.2.3. Multifocality (3 Parameters)

A connected component approach was used to detect the different stand-alone tumor foci, and the diameter of each was computed. After identification of the biggest tumor piece, the number of additional independent foci was computed. Only satellite foci away from the biggest foci by a minimum distance of at least 5.0 mm were taken into consideration. A Boolean indicates the multifocality status, and the total amount of independent foci are reported. In addition, the largest minimum distance between the biggest foci and any satellite foci is reported. In the case of a unifocal tumor, a distance value of −1.0 mm is reported.

#### 3.2.4. Resectability (2 Parameters)

The expected residual tumor volume and the expected resectability index were computed following the methodology described in [43]. In the original work, a resection heat map was built for each hemisphere. As such, based on the hemisphere with the highest tumor overlap percentage, the proper heat map was used for the feature computation. The expected resectable volume, expressed in milliliters, was obtained by integrating the resection probabilities from the heat map for the voxels masked by the tumor segmentation. The expected residual tumor volume was computed as the difference between the preoperative segmented tumor volume and the expected resectable volume (expressed in milliliters). Finally, the expected resectability index was generated from the division of the expected resectable volume by the preoperative segmented tumor volume, ranging from [0,1] on a continuous scale. A value of zero represents a nonresectable tumor, whereas a value of one represents a completely resectable tumor.

#### 3.2.5. Cortical Structure Location Profile (87 Parameters)

**Figure 2:**
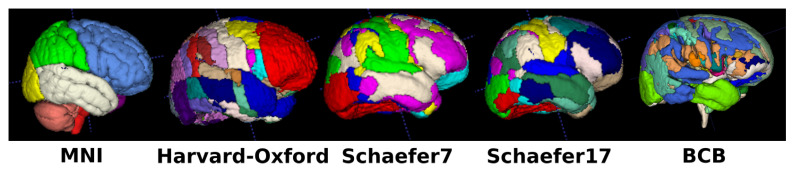
Illustration of the different atlases used for feature computation, all expressed in the MNI (Montreal Neurological Institute) space. Cortical structures atlases: MNI, Harvard-Oxford, Schaefer7, and Schaefer17. Subcortical structures atlases: BCB (Brain Connectivity Behaviour).

The following three atlases were used: MNI [44], Harvard-Oxford [45,46,47,48], and Schaefer [49,50] (cf. the four leftmost illustrations in Figure 2). From the MNI structural atlas, a total of 15 different structures are represented, often with the distinction between left and right hemisphere. In the Harvard-Oxford atlas, 48 cortical structures are introduced from Desikan’s brain parcellation based on anatomy. Finally, in the Schaefer atlas, only the 7 and 17 versions were used with the corresponding number of network classes from the 400 parcels, based on functional connectivity using resting-state functional MRI. For each structure of each atlas, the ratio between the tumor volume overlapping the structure and the total tumor volume was computed and expressed as a percentage.

#### 3.2.6. Subcortical Structure Location Profile (2×68 Parameters)

The atlas from the Brain Connectivity Behaviour (BCB) group [51] was used (cf. rightmost illustration in Figure 2). A total of 68 subcortical structures, often disambiguated between left and right hemispheres, are featured for a total of 40 unique structures agnostic of laterality. The atlas being probabilistic, threshold values were necessary to compute the distances and overlaps between the two binary masks. For the BCB atlas, computations were performed using a probability threshold set to 0.5.

For each subcortical structure of each atlas, the ratio between the tumor volume overlapping the structure and the total tumor volume was computed and expressed as a percentage. For structures not overlapping with the tumor, the minimum distance between the two is reported in millimeters and computed using the 95th percentile Hausdorff distance metric.

### 3.3. Proposed Software and Standardized Reporting

**Figure 3:**
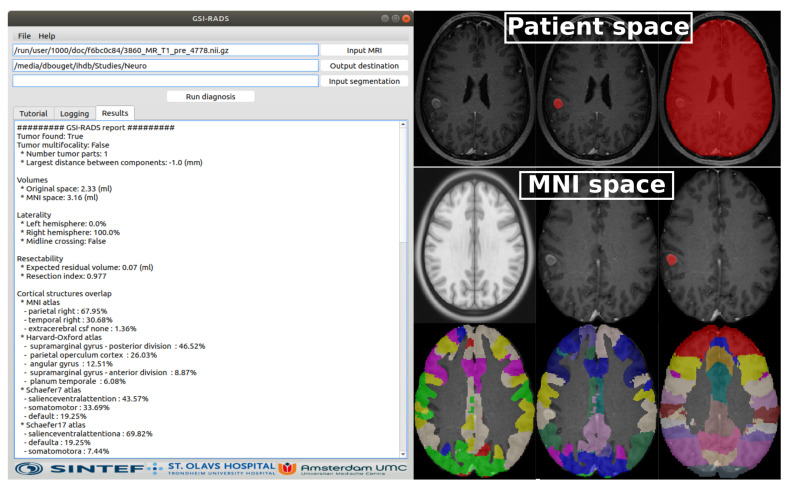
Illustration of the proposed GSI-RADS. The GUI (Graphical User Interface) is presented to the left with a standardized report. To the right, the different outputs both in the patient and MNI space are illustrated. In the bottom row, the Schaefer7, Schaefer17, and Harvard-Oxford cortical structures are respectively shown.

The developed software, GSI-RADS, enables the user to run the tumor segmentation and automatic feature computation as described above. In the first version, only models trained with the AGU-Net architecture are supported, and future versions will also handle nnU-Net models. The software is freely available at https://github.com/SINTEFMedtek/GSI-RADS, accessed on 16 September 2021. The software, coming with a minimalistic Graphical User Interface (GUI), was developed using Python 3.6, PySide2 v5.15.2, and only uses the Central Processing Unit (CPU) for the various computations. The compatibility for use with Windows (≥10), macOS (≥high-sierra), and Ubuntu Linux (≥18.04) was tested, and a separate executable was created for each operating system. The software expects the MRI volume in NIfTI or DICOM format as the input and a location to save the results. In addition, the possibility to provide the corresponding tumor segmentation mask was left open (in NIfTI format) to skip directly to the tumor feature computation and reporting step. An illustration of the GUI is provided in Figure 3. For completeness, the source code is available with options for running the different methods without a GUI, directly through the command line interface. In that configuration, Graphics Processing Unit (GPU) support is enabled for computation.

The standardized reporting is first available as text format where the different features are grouped following the same categories as above. For the cortical and subcortical structure features, only the instances with a non-null overlap percentage are reported to avoid visual overload. To complement the reporting and give the possibility for follow-up statistical studies, the complete set of computed features are also provided in comma separated value format (i.e., .csv). In addition to the two above-mentioned reports, the following files are provided in NIfTI format to enable a visual assessment of the results. In the original patient space are provided the following: tumor and brain segmentation masks and the cortical structures masks. Furthermore, the original MRI volume and corresponding tumor segmentation mask, resulting from the registration process, are also provided in the MNI space.

## 4. Validation Studies

In the validation studies, we focused on assessing automatic segmentation performance. The clinical validity and relevance for the feature extraction and standardized reporting was addressed and reported in a recent study [32]. For the current task of pixelwise segmentation, only two classes were considered: tumor tissue and nontumor tissue. In that sense, a positive pixel is a pixel exhibiting tumor tissue, whereas a negative pixel is a pixel exhibiting background or normal tissue.

### 4.1. Protocols

Given the relatively large size of our dataset and important GPU memory and training requirements to perform multiple validation studies, the following three protocols were applied.

#### 4.1.1. Leave-One-Hospital Out

A three-way split at the hospital level was performed on the GS1 subset. The MRI volumes from one hospital constituted the validation fold; the MRI volumes from a second hospital constituted the test fold; the remaining MRI volumes constituted the training fold. As such, each hospital was used in turn as the test set in order to properly assess the ability of the different models to generalize. The two hospitals held out for each fold are reported in Table 1.

#### 4.1.2. Custom Validation

To perform investigations in a reasonable amount of time, while still being relevant, a custom validation protocol was set over the GS1 subset. Instead of training 13 models as in the above protocol, we decided to only train models from the following three representative folds: 1, 10, and 13.

#### 4.1.3. BraTS External Validity

The training fold from the 2020 BraTS challenge dataset, constituting our GS2 subset, was used as an external test fold. Models trained on the GS1 subset using the custom validation protocol were recursively applied on the GS2 subset and the results averaged.

### 4.2. Metrics and Measurements

In addition to the standard pixelwise metrics, the study was reinforced with patientwise, as well as speedwise metrics. Pooled estimates [52], computed from each fold’s results, are reported for each measurement with either mean or mean and standard deviation.

#### 4.2.1. Pixelwise

To quantify the semantic segmentation performance, pixelwise metrics were computed over the full 3D MRI volume between the ground truth tumor annotation and a thresholded binary tumor prediction mask. We chose to report: (i) the Dice score measuring the volume similarity as the ratio between the intersection and sum of volumes and (ii) the 95th percentile Hausdorff distance (HD95) assessing the boundary delineation quality (i.e., contours). The 95% version was used to make measurements less sensitive and more robust to small outliers.

Each pixelwise metric was computed between the ground truth and a binary representation of the probability map generated by a trained model. The binary representation was computed for ten different, equally spaced, Probability Thresholds (PTs) in the range [0,1].

#### 4.2.2. Patientwise

To quantify the detection performances, we computed the following patientwise performances: recall, precision, and F1-score. In addition, we report the Dice score only for the true positive tumors (i.e., correctly detected gliomas), noted as Dice-TPs. Finally, the average number of erroneous detections per patient was calculated as False Positives Per Patient (FPPPs).

A connected components approach coupled to a pairing strategy was employed to compute the patientwise metrics. A minimum threshold of 50 voxels was set, and objects below that limit were discarded. A Dice score Threshold (DT) at the patient level was used, with a selected value of 0.25, to determine if a tumor had been sufficiently segmented to be considered a true positive.

#### 4.2.3. Speedwise

To quantify the runtime performances, the computation speed was measured for the different steps of the pipeline in order to generate a standardized report. The pipeline is represented by: (i) segmentation of the brain, (ii) segmentation of the glioblastoma, (iii) registration to the MNI space, and (iv) computation of tumor features and saving of the report. The segmentation tasks included preprocessing, model initialization, inference, and reconstruction in the original space.

A typical MRI volume was chosen, and speeds were averaged after sequentially repeating an experiment 10 times, with CPU usage only.

### 4.3. Experiments

(i) Architecture comparison: Using the leave-one-hospital-out protocol, the two considered architectures were compared in terms of segmentation performance. Results are reported over both the entire GS1 subset in addition to separately for each fold. A further performance comparison was performed when grouping the tumors based on volume (i.e., <3 mL and ≥3 mL) and focality (i.e., unifocal/multifocal).

(ii) External validity: The GS2 subset, from the BraTS challenge, was used as a benchmark for comparing segmentation performances using the three models, as described in the custom validation protocol, for each of the two considered architectures. For an additional comparison, results over the BraTS validation set from the H${}^{2}$NF-Net [26], a top-ranked method from the BraTS challenge edition 2020, are reported.

(iii) Preprocessing impact: The comparison of the segmentation performances among the different preprocessing strategies, following the custom validation protocol was made. For completeness, the performances over the BraTS challenge training set (i.e., GS2 subset) were also computed.

(iv) Speed performance study: To assess the feasibility for deployment and use in clinical practice, two representative high-resolution MRI volumes were selected for the study.: first, a sample of 256×256×176 voxels with 1.0×1.0×1.0 mm${}^{3}$ spacing (Sample1); second, a sample with a double-axial resolution of 512×512×261 voxels with 0.51×0.51×1.02 mm${}^{3}$ spacing (Sample2). Speed performances were acquired and compared using a model trained with the AGU-Net architecture and the P2 preprocessing scheme.

(v) Inter-rater variability: A subset of the MRI volumes from the AMS hospital (i.e., 20 patients) was annotated once by 8 different annotators (4 experts and 4 novices). For both architectures, segmentation performances are reported over this subset using the model corresponding to fold 13, where the AMS hospital belongs to the test set. In addition, multiple consensus ground truths were created for each of the 20 patients using a majority voting approach (i.e., all raters, novice raters, and expert raters). For a voxel to belong in the consensus ground truth, at least half of the raters must have annotated it.

## 5. Results

### 5.1. Implementation Details

Results were obtained using two different machines: (i) Intel Xeon W-2135 CPU @3.70 GHz x 12, 64 GB of RAM, NVIDIA Quadro P5000 (16 GB), and a regular hard-drive and (ii) AMD Ryzen 9 3900X @3.80 GHz, 64 GB of RAM, NVIDIA RTX 3090 (24 GB), and an NVMe SSD. The implementation was performed in Python 3.6 using TensorFlow v1.13.1 for the AGU-Net architecture, whereas Python 3.8.8 and PyTorch v1.9.0 were used for the nnU-Net architecture.

### 5.2. Architecture Comparison

The average segmentation performance for the two main architectures, averaged over the 13 folds, is reported in Table 2. The nnU-Net architecture reached higher Dice and recall values, benefiting from the use of the high-resolution MRI volume as the input. The additional voxels enabled a better pixelwise refinement, hence an overall Dice score of 86.88%, more than 4% higher than with the AGU-Net architecture. From its patchwise design and access to more local information, nnU-Net was hence also able to reach a near-perfect patientwise recall with 98.11%. Out of the 1594 patients featured in the GS1 subset, only 30 cases were not identified or had a Dice score below 25% with nnU-Net, compared to 75 cases with AGU-Net. As indicated by the Dice-TP score, the pixelwise segmentation quality gap between the two methods was reduced to 2% when considering only the true positive cases. In contrast, the AGU-Net architecture design, leveraging the whole brain at once, was able to reach a superior precision and greatly reduce the amount of false positive tumor detection. As such, a patientwise precision of 97.37% could be reached, which was 7% higher than with nnU-Net.

Similarly, and thanks to the reduced rate of false positive detections, an average 95th percentile Hausdorff distance of 6.09 mm was obtained with AGU-Net, compared to the 10.59 mm from nnU-Net.

**Figure 4:**
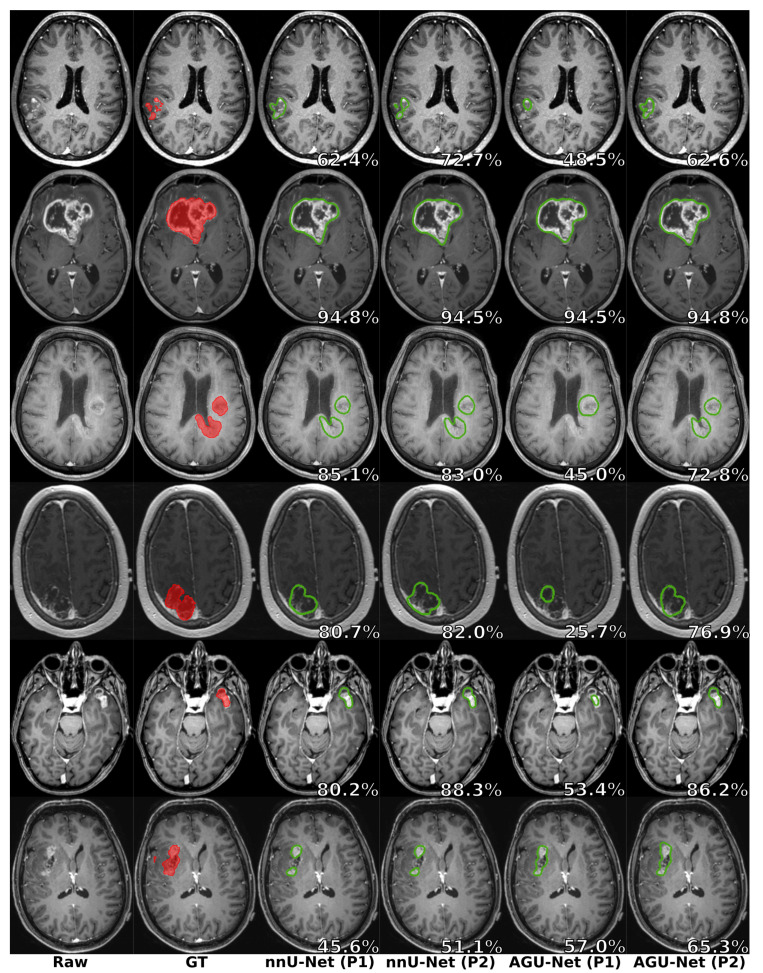
Segmentation comparison between the manual ground truth (overlaid in red) and the predictions (outlined in green) from the two architectures and with the two different preprocessing schemes. One patient is featured per row, and the overall raw Dice score is reported in white (best viewed digitally and in color).

Considering unifocal glioblastomas, the performances were quite similar to the overall average performances. However, a decrease in the performances can be noticed for the multifocal glioblastomas, with a Dice score lower by 4% on average. The increased amount of satellite foci to detect and segment, often with a considerably smaller volume than the main foci, explained the difference in the Dice score. Finally, a similar conclusion can be drawn for the glioblastomas with a volume smaller than 3 mL. A noticeable drop in the Dice score can be witnessed for the nnU-Net architecture, but a patientwise recall of 84% could still be reached thanks to its design of leveraging local information at a high resolution. Such small tumors, represented by a tiny voxel fraction of an MRI volume, were even more difficult to detect and segment for the AGU-Net architecture, which further downsampled the input MRI volume. As a consequence, the patientwise recall could only reach 61% for the smallest glioblastomas, while nearing 98% for the rest of the tumors and comparable to the corresponding nnU-Net patientwise recall of 99%. Visual comparisons are provided in Figure 4 between the two architectures for six patients, one per row.

Detailed segmentation performances for each hospital featured in the GS1 dataset, using both architectures, are reported in Table 3. Across each hospital, the same global trend can be witnessed with nnU-Net providing higher Dice and recall scores while AGU-Net reached a higher precision, F1-score, and HD95. The performances were also stable across the different hospitals, only with a noticeable drop in the Dice score for the St. Olavs hospital (STO). This deviation can be explained by the number of patients featured, the largest of the thirteen hospitals by a huge margin, hence greatly reducing the amount of samples used for training the corresponding model and, as such, its ability to generalize. To conclude, the results indicate that our custom validation protocol, training models and reporting results only for the three hospitals, will provide good insights representative of the overall expected performances.

### 5.3. External Validity

The average results over the GS2 subset, obtained from running inference with the three trained models as described in the custom validation protocol, are reported in Table 4. Unfortunately, segmentation performance results over the training set from the BraTS challenge were difficult to come by. Therefore, the selected baseline results from H${}^{2}$NF-Net [26] were computed over the validation set, and simply for the Dice score and 95th percentile Hausdorff distance.

Considering exclusively those two metrics, the nnU-Net models trained on the GS1 subset transferred well to the GS2 subset with similar performances to the baseline. Conversely, the AGU-Net models struggled with a 3% lower Dice score than nnU-Net, which can be explained by the lower patientwise recall, although balanced by a 2% better Dice-TP score. Given the innate state of MRI volumes featured in GS2 to have already undergone a brain-clipping operation, the performances from the models trained on GS1 were surprisingly positive. Following the P1 preprocessing, where the whole head was kept intact, the field-of-view difference between MRI volumes from GS1 and GS2 was quite noticeable and could have led to larger differences in segmentation performance.

### 5.4. Preprocessing Impact

For both architectures, the segmentation performances when using the different preprocessing schemes, and over both data subsets, are reported in Table 5. Interestingly, an overall performance improvement was noticeable when using the second preprocessing scheme, for both architectures and over both data subsets. The brain-masking operation increased the field-of-view over the brain and discarded irrelevant regions inside the MRI volume where glioblastomas cannot exist. The operation was extremely beneficial for the nnU-Net architecture, as it reduced the amount of potential confusing locations, hence enabling an impressive boost in precision. The patientwise precision was lifted up to 98%, from 82%, when using the first preprocessing scheme over GS1. The operation was similarly beneficial for the AGU-Net architecture since a virtual resolution increase resulted from discarding the regions outside the brain. With a limitation to the downsampled input resolution weakness, the patientwise recall value and Dice score were boosted.

As a result, the performance gap between nnU-Net and AGU-Net was closed when using the second preprocessing scheme. A Dice score slightly below 90%, a patientwise F1-score of 98.7%, and an HD95 slightly below 4.0 mm could be reached on average with both architectures.

Regarding the performances over the second subset, the second preprocessing scheme rendered the training samples more similar to the available MRI volumes in GS2. In addition to the benefits listed above for each architecture, the performances reached were higher than with the first preprocessing scheme, and comparable to the performances over GS1.

### 5.5. Speed Performance Study

The speedwise results regarding the different steps of standard report generation are summarized in Table 6 and were obtained with Machine (i) described in Section 5.1. Overall, between 4.0 min and 5.5 min were required in total to fully process one patient’s MRI volume and generate the report. The variation in runtime was based on the input volume resolution, as larger MRI volumes necessitated longer processing. Segmentation of the glioblastoma was the fastest of all steps, performed in around 30 s at most for large MRI volumes, independent of the preprocessing scheme. Indeed, the segmentation of the brain was a required core step, which must be performed before registration to the MNI space. As such, the brain did not need to be segmented a second time as part of the P2 preprocessing scheme. Unlike every other step of the pipeline, the tumor feature computation was not impacted by the input volume resolution, requiring 2.3 min on average.

### 5.6. Inter-Rater Variability

For the 20 patients from the AMS hospital, the inter-rater manual segmentation variability is reported in Table 7. For both architectures, the Dice scores obtained over the ground truth were within 1% of the scores obtained after majority voting across the eight raters. Overall, this agreement indicates the quality of the manual ground truth used in the study and the trustworthiness of the trained models. Another indication from the table was the apparent better manual segmentation quality from the expert raters compared to the novice raters. The average Dice scores for the expert raters was close to the manual ground truth and about 1–2% higher than for the novice raters.

**Figure 5:**
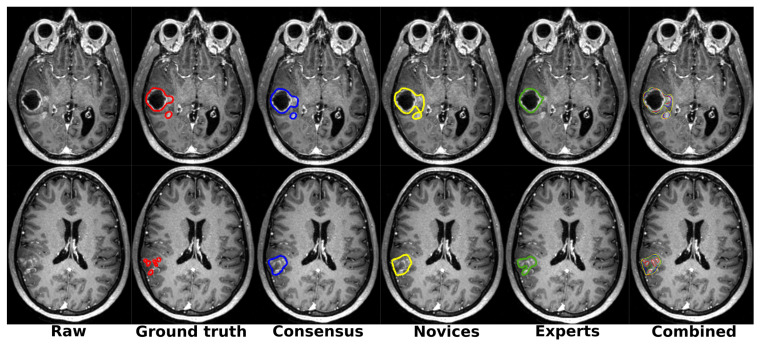
Illustration of the manual segmentation differences between the different raters, one patient per row. Color code: red is the ground truth; blue is the majority voting across all raters; yellow is the majority voting across novice raters; green is the majority voting across expert raters (best viewed digitally and in color).

The substantial patientwise Dice score variations across raters, reported in the three rightmost table columns, demonstrated the difficulty in manually segmenting glioblastomas. For two patients, the variations between the different groups of raters are illustrated in Figure 5.

## 6. Discussion

In this study, an in-depth analysis and assessment of glioblastoma segmentation performances was carried out using two state-of-the-art architectures: nnU-Net and AGU-Net. The main finding to consider is the high quality of glioblastoma pixelwise segmentation and detection performances from the sole use of a gadolinium-enhanced T1-weighted MRI volume. Both presented architectures exhibited a proper ability to generalize, as shown by the cross-hospital study and external validity study over the BraTS challenge dataset. In addition, a fine-tuning of the preprocessing scheme was identified as key to further improvement, and in the case of glioblastomas, a brain-masking operation appeared to be extremely relevant.

Stemming from their inherent design characteristics, both the nnU-Net and AGU-Net architectures exhibited a complementary behavior. The use of a patchwise strategy over a high-input-resolution MRI volume with nnU-Net enabled the trained models to reach higher recall values. From its magnified usage of local information, even the smallest glioblastomas with a volume below 3 mL or satellite foci in the case of multifocal tumors, were better segmented. For larger tumors, the gap in the Dice score performances between the two architectures was greatly reduced. The access to a higher level of local details with nnU-Net enabled generating slightly more refined segmentations around the tumors’ borders. On the other hand, the AGU-Net architecture leveraged an entire MRI volume, only in a greatly downsampled manner (i.e., low resolution). Having access to global contextual information, the trained models showed an improved ability to disambiguate between glioblastomas and neighboring contrast-enhancing structures (e.g., blood vessels). Subsequently, a notably reduced number of false positive segmentations per patient was visible, leading to near-perfect precision performance. At the moment, a unique convolutional neural network architecture able to combine the strengths of both nnU-Net and AGU-Net is impossible to train, as it does not fit on GPU memory. Yet, the use of MRI volumes at their native resolution together with the joint generation of better global context features and the preservation of local information is key to further improvements. A favored state-of-the-art alternative requires training the models sequentially, ideally with different designs, and combining them in a cascade or ensemble fashion to virtually benefit from the previously stated joint generation.

Nevertheless, advancing or fine-tuning the training preprocessing schemes represents another alternative to improve the performances and was shown in this study to reduce the gap between the two considered architectures. Preprocessing schemes being a collection of operations intending to condition and normalize the data in order to facilitate the training task, any combination of operations is possible. Given the training time duration, the focus was brought towards two preprocessing schemes only, facing technical and feasibility limitations for investigating more. A simple, fast-to-compute, and computationally efficient scheme was first designed with the minimum amount of operations (P1). An MRI volume could be processed in <15 s, and baseline results could be obtained, from setting up such a scheme. Then, in order to generate optimal segmentations over the BraTS challenge dataset (GS2), the second preprocessing scheme was mandatory (P2). The brain-masking operation was a core component of the second scheme and represented the main reason for the boost in segmentation performances over both subsets. While potentially detrimental for the segmentation of other brain tumor types (e.g., meningioma), glioblastomas are located well within the brain boundaries and will not suffer from such an operation. Finally, the corresponding computational overhead was reasonable, with an additional 60 to 90 s required for processing one MRI volume. Using a more recent or optimized method for performing brain segmentation should even reduce the required runtime to <15 s.

More advanced and complex operations were considered such as N4 bias correction or atlas-based registration to a common space. Yet, the computational overhead for such methods can be counted in minutes for an unlikely benefit (cf. Supplementary Material Section S4.3). The tradeoff between a potential increase in segmentation performances and processing time does not justify their use at the time. Moreover, an aim of this study was to provide easily applicable solutions, designed for wide-spread adoption. As such, less complex and computationally intensive workflows are to be favored.

From the extensive validation studies performed in this study, the ability to generalize from the trained models of both architectures was demonstrated. Similar segmentation and detection performances were obtained for the different models trained over the 13 hospitals, tallying close to 1600 patients. The same models were shown to be equally robust when applied to the BraTS challenge dataset, further confirming the external validity. Interestingly, a drop in performances was identified for glioblastomas with a total volume below 3 mL, far below average. Given the small amount of voxels representing the tumor, the Dice metric was in this case extremely sensitive to slight variations in segmentation quality. Further investigations are essential to design better or smarter architectures able to bring the Dice and recall values to the same height as for their ≥3 mL counterpart. Alternatively, loss functions and validation metrics should better combine the 95th percentile Hausdorff distance or F1-score together with the Dice score, to better convey overall performance. Overall, the high-quality segmentation reached by these models, close to the manual ground truth, highlights the great potential for use in automatic clinical support device systems, such as GSI-RADS.

The generation of automatic glioblastoma segmentation, and subsequently of tumor features, was faster, less tedious, and more reproducible than from a manual process. In our previous study [32], an excellent agreement between tumor features extracted from the manual ground truth and automatic segmentations was identified, from models trained using the AGU-Net architecture and the P1 preprocessing scheme. In this study, the automatic segmentation performance was further improved, and the models’ robustness across 14 different MRI data sources was showcased. The standard reports for glioblastoma surgery generated by our developed GSI-RADS software showed the potential for practical implication. Already now, such reports could serve educational or training purposes for neurosurgical trainees and could speed up the learning curve. Furthermore, the segmentation models could be employed by other institutions or research groups in order to generate annotated datasets in a less time-consuming way. To facilitate the spreading of the standard methods, the GSI-RADS software is freely available online together with the best segmentation models. The clinical relevance and potential advantages in practice of this work were covered in-depth in our previous study [32]. With automated segmentations in excellent agreement with manual annotations, stable and trustworthy tumor features can be computed, potentially relevant for neurosurgical planning and reporting. While improved patient outcomes cannot be expected from a standardized reporting in itself, more accurate data-driven decisions can be taken from the preoperative imaging. Indirectly, a better communication between neurosurgeons and teams can be achieved, especially around complex surgical cases.

Future efforts could be for one to further improve the pixelwise segmentation and patientwise detection quality by designing better multiscale architectures or more complex loss functions. Alternatively, other tumor compartments could be segmented, such as necrotic or ischemic tissue, hemorrhage, and cyst fluid; requiring access to T2 and FLAIR MRI sequences to complement the T1-weighted MRI volumes. Lastly, reliable tumor segmentation should not be exclusive to pre-operative MRI volumes. Segmentation over time and at different stages of the disease is extremely valuable to provide standardized reports of postsurgical evaluation and treatment response assessment. Regarding tumor feature extraction, further work is for example required on the quantification of different aspects of multifocality, as well as infiltration and disconnection of white matter pathways. A comparison between extracted features and patient outcomes bears potential, but would likely require a larger dataset to be conclusive. Finally, an extension of the GSI-RADS software to other pathologies, such as low-grade nonenhancing glioma, meningioma, and brain metastasis, is to be considered.

## 7. Conclusions

The proposed GSI-RADS software generates standardized reports from an automatic tumor segmentation, from which the extracted features include volume, laterality, multifocality, location, and resectability. The in-depth and extensive validation studies across 14 different data sources proved the high quality, robustness, and ability to generalize of the trained segmentation models. The online and free access to the complete software should encourage its use for relevant neurosurgical purposes.

## Figures and Tables

**Table 1:** Hospitals retained as part of the validation and test sets, per fold, with the leave-one-hospital out protocol.

Fold	1	2	3	4	5	6	7	8	9	10	11	12	13
Val.	HAG	MIL	ZWO	VIE	ALK	PAR	SLO	STO	SFR	GRO	UTR	AMS	TIL
Test	TIL	HAG	MIL	ZWO	VIE	ALK	PAR	SLO	STO	SFR	GRO	UTR	AMS

**Table 2:** Segmentation performances obtained with the two considered architectures using the P1 preprocessing scheme, averaged over the 13 folds of the GS1 subset. For each category, the upper row corresponds to nnU-Net and the lower row to AGU-Net. The tumor volume threshold for the small and large categories was set to 3 mL.

Category	Pixelwise			Patientwise (PW)			
	Dice	Dice-TP	HD95	FPPP	F1	Recall	Precision
All	86.88±14.49	88.38±01.65	10.59±27.94	0.26±0.29	93.88±04.81	98.11±01.23	90.37±08.50
	82.14±20.28	86.04±01.49	06.09±10.82	0.05±0.04	96.27±01.39	95.27±02.44	97.37±02.04
Unifocal	87.74±13.77	89.13±01.60	10.74±28.77	0.26±0.29	93.47±05.34	98.27±01.36	89.53±09.22
	83.69±18.83	86.98±01.52	04.98±09.41	0.04±0.04	96.87±01.38	96.03±01.95	97.77±01.95
Multifocal	84.12±16.08	85.94±02.16	10.10±24.69	0.25±0.33	95.12±04.66	97.60±03.36	93.09±07.92
	77.15±23.40	82.86±03.34	09.66±13.69	0.09±0.09	94.26±03.05	92.80±06.68	96.10±03.37
Small	63.44±28.01	73.66±08.73	31.56±43.84	0.53±0.66	81.92±11.59	84.74±10.10	81.67±16.66
	45.21±34.75	71.92±08.18	09.11±16.15	0.09±0.11	72.06±15.09	61.01±20.55	93.62±09.15
Large	88.76±10.60	89.41±01.45	08.95±25.34	0.24±0.27	94.77±04.56	99.18±00.78	91.04±08.03
	85.11±14.89	86.75±01.75	05.84±10.14	0.05±0.05	97.79±01.32	98.02±01.22	97.58±02.21

**Table 3:** Hospitalwise segmentation and detection performance comparison between the two main architectures. For each hospital, the upper row represents nnU-Net and the lower row represents AGU-Net.

Hospital	Pixelwise			Patientwise (PW)			
	Dice	Dice-TP	HD95	FPPP	F1	Recall	Precision
TIL	90.37±10.42	90.95±07.53	09.33±28.80	0.19	95.93	99.34	92.74
	87.55±13.74	89.22±07.00	04.63±06.51	0.02	98.35	98.03	98.67
HAG	89.23±11.65	89.89±06.62	22.59±45.08	0.76	84.39	99.02	73.52
	84.68±18.38	88.10±07.07	04.57±09.82	0.01	97.61	96.11	99.15
MIL	90.95±06.60	90.95±06.60	03.89±11.70	0.09	98.13	100.0	96.33
	85.10±17.24	87.42±10.17	04.31±07.76	0.02	97.98	97.33	98.64
ZWO	89.09±11.97	90.28±06.57	14.57±32.33	0.33	92.39	98.61	86.91
	82.26±18.28	84.61±12.04	06.19±08.75	0.09	96.46	97.22	95.71
VIE	91.70±03.73	91.70±03.73	05.68±16.43	0.12	97.35	100.0	94.83
	84.56±15.97	86.64±08.97	06.00±09.92	0.02	98.17	97.59	98.76
ALK	87.98±10.62	87.98±10.62	24.95±43.38	0.84	86.55	100.0	76.30
	81.32±25.22	88.29±08.69	03.22±03.12	0.05	95.00	92.10	98.09
PAR	88.91±12.14	90.13±06.30	04.56±11.81	0.06	97.62	98.64	96.62
	80.61±22.08	86.11±08.37	04.43±04.15	0.01	96.16	93.24	99.28
SLO	88.21±11.61	89.56±06.98	05.30±11.81	0.20	93.88	97.95	90.13
	82.54±19.69	86.05±10.11	10.93±25.20	0.10	95.65	95.91	95.39
STO	82.14±20.19	85.75±11.93	09.73±24.63	0.15	94.39	95.38	93.43
	77.78±26.29	85.29±12.22	06.35±09.83	0.05	93.83	90.68	97.20
SFR	87.25±13.47	88.57±08.19	06.71±20.78	0.09	97.48	98.50	96.49
	82.84±17.28	84.73±12.04	05.70±08.39	0.07	97.36	97.76	96.96
GRO	87.15±11.61	87.15±11.61	30.27±50.14	0.93	84.42	100.0	73.04
	85.46±13.95	86.37±11.18	08.45±16.69	0.18	95.42	98.83	92.24
UTR	87.40±10.87	88.36±06.39	04.95±12.57	0.14	96.62	98.83	94.50
	82.60±13.82	84.03±08.81	06.62±09.49	0.10	97.13	98.24	96.05
AMS	87.93±09.93	87.93±09.93	09.73±26.30	0.34	91.82	100.0	84.87
	83.39±17.22	85.82±10.70	07.56±15.36	0	98.42	96.90	100.0

**Table 4:** Segmentation performances obtained over the GS2 subset with the two considered architectures and a baseline. The results are averaged from the three models trained following the custom validation protocol with P1 preprocessing.

Arch.	Pixelwise			Patientwise (PW)			
	Dice	Dice-TP	HD95	FPPP	F1	Recall	Precision
H${}^{2}$NF-Net	85.46	-	4.18	-	-	-	-
nnU-Net	84.07±13.76	85.44±0.22	4.07±4.84	0.06±0.01	97.73±0.29	98.29±0.27	97.19±0.41
AGU-Net	81.47±23.31	87.27±0.21	5.67±8.60	0.11±0.06	93.95±0.90	93.17±0.96	94.83±2.84

**Table 5:** Segmentation performances obtained with the different architectures and preprocessing schemes, averaged from the three models of the custom validation protocol, over the two data subsets.

Configuration	Pixelwise			Patientwise (PW)			
	Dice	Dice-TP	HD95	FPPP	F1	Recall	Precision
nnU-Net/GS1/P1	88.66±11.52	89.36±1.30	08.52±25.58	0.19±0.09	95.43±2.39	99.21±0.61	92.06±4.38
nnU-Net/GS1/P2	89.14±11.04	89.82±1.14	03.90±10.23	0.06±0.02	98.73±0.56	99.21±0.61	98.25±0.55
AGU-Net/GS1/P1	85.08±15.48	86.80±1.91	05.76±10.09	0.03±0.03	98.15±0.31	97.91±0.66	98.41±1.24
AGU-Net/GS1/P2	87.42±13.91	88.70±1.19	03.86±6.70	0.02±0.01	98.72±0.57	98.43±0.70	99.01±0.76
nnU-Net/GS2/P1	84.38±15.98	86.74±1.08	18.11±30.04	0.44±0.13	89.90±2.29	97.15±0.16	83.74±3.93
nnU-Net/GS2/P2	84.07±13.76	85.44±0.22	04.07±4.84	0.06±0.01	97.73±0.29	98.29±0.27	97.19±0.41
AGU-Net/GS2/P1	81.47±23.31	87.27±0.21	05.67±8.60	0.11±0.06	93.95±0.90	93.17±0.96	94.83±2.84
AGU-Net/GS2/P2	87.04±16.10	89.38±0.46	04.44±8.06	0.14±0.05	95.56±0.98	97.38±1.60	93.84±2.03

**Table 6:** Speed performances for the segmentation, feature extraction, and overall standardized report generation. Results were averaged after 10 consecutive runs over two representative MRI volumes, using CPU support only.

	Brain Segmentation (s)	Registration (s)	Tumor Segmentation (s)	Features Computation (s)	Total (s)	Total (m)
Sample1	54.33±1.47	40.80±0.93	14.75±0.43	144.36±2.21	254.28±3.59	4.24±0.06
Sample2	99.43±1.37	55.38±0.27	32.99±0.31	139.36±0.94	327.17±1.49	5.45±0.02

**Table 7:** Inter-rater Dice score variability, averaged over the 20-patient subset from the AMS hospital, for the two architectures and preprocessing schemes. The ∆ columns indicate the average patientwise Dice score variations across the different categories of raters.

Arch.	Pre.	Ground Truth	Consensus	Novices	Experts	∆Total	∆Novices	∆Experts
nnU-Net	P1	87.23±12.30	88.90±13.76	86.61±15.69	87.38±12.30	10.04	6.13	8.73
	P2	88.80±10.15	89.05±14.34	86.22±16.81	87.88±14.49	10.10	7.11	7.74
AGU-Net	P1	80.28±23.99	80.38±25.89	79.06±26.35	80.14±25.29	6.88	4.46	4.84
	P2	85.80±14.90	87.15±15.85	84.84±18.20	86.28±16.49	9.84	5.31	8.72

## Data Availability

The manual segmentation data can be found as publicly archived dataset (https://doi.org/10.17026/dans-xam-j5aw, accessed on 16 September 2021). The open-access software, trained models, and source code can be found as a public archive (https://github.com/SINTEFMedtek/GSI-RADS, accessed on 16 September 2021).

## Supplementary Materials

The following are available at https://www.mdpi.com/article/10.3390/cancers13184674/s1, Table S1: Overview of the distribution of patients per hospital in the GS1 subset, Table S2: Hospitalwise segmentation and detection performances comparison between the two main architectures. For each hospital, the upper row represents nnU-Net and the lower row represents AGU-Net, Table S3: Segmentation performances obtained with the two considered architectures using the P1 preprocessing scheme, averaged over the 13 folds of the GS1 subset. For each category, the upper row corresponds to nnU-Net and the lower row to AGU-Net. The tumor volume threshold for the small and large categories was set to 3 mL, Table S4: Segmentation performances obtained over the GS2 subset with the two considered architectures and a baseline. The results are averaged from the 3 models trained following the custom validation protocol with P1 preprocessing, Table S5: Segmentation performances obtained with the different architectures and preprocessing schemes, averaged from the three models of the custom validation protocol, over the two data subsets, Table S6: Segmentation performances obtained with the different architectures and preprocessing schemes, averaged from the 3 models of the custom validation protocol, on the specified data subset.
