# Supplementary material for: Glioblastoma Surgery Imaging–Reporting and Data System: Validation and Performance of the Automated Segmentation Task
Source: Cancers (Basel). 2021 Sep 17;13(18):4674. doi: 10.3390/cancers13184674 (PMC8465753; doi:10.3390/cancers13184674)
Supplement: Supplementary file 1 [file cancers-13-04674-s001.zip › cancers-1342385-supplementary.pdf]

Supplementary Materials: Glioblastoma Surgery Imaging–Reporting and Data System: Validation and Performance of the Automated Segmentation Task

David Bouget *, Roelant S. Eijgelaar, André Pedersen, Ivar Kommers, Hilko Ardon, Frederik Barkhof, Lorenzo Bello, Mitchel S. Berger, Marco Conti Nibali, Julia Furtner, Even Hovig Fyllingen, Shawn Hervey-Jumper, Albert J. S. Idema, Barbara Kiesel, Alfred Kloet, Emmanuel Mandonnet, Domenique M. J. Müller, Pierre A. Robe, Marco Rossi, Lisa M. Sagberg, Tommaso Sciortino, Wimar A. Van den Brink, Michiel Wagemakers, Georg Widhalm, Marnix G. Witte, Aeilko H. Zwinderman, Ingerid Reinertsen, Philip C. De Witt Hamer and Ole Solheim

1. Dataset

The distribution of patients per center, from the GS1 subset, is reported in Table S1.

Table S1: Overview of the distribution of patients per hospital in the GS1 subset.

Hospital	TIL	HAG	MIL	ZWO	VIE	ALK	PAR	SLO	STO	SFR	GRO	UTR	AMS
Samples	153	103	75	72	83	38	74	49	459	134	86	171	97

2. Method

2.1. Segmentation

For the AGU-Net architecture, a third preprocessing P3 has been investigated as a preliminary study. The difference from P2 is the additional registration to MNI space.

1. P3: (i) resampling to an isotropic spacing of 1 mm^3 using spline interpolation order 1, (ii) brain segmentation and brain-masking, (iii) registration to MNI space using the SyN method from ANTs, and (iv) zero-mean normalization of intensities.

In addition, a variant of the architecture was also investigated, called AGU-Net-W, with an increased number of total parameters from a wider design.

Architecture design:

Five levels and $[32, 64, 128, 256, 512]$ as filter sizes, and an input size set to $128 \times 128 \times 144$ voxels. The remainder of the design is similar to AGU-Net.

2.2. Clinical features computation

For the cortical and subcortical structures, the exhaustive lists of included structures are listed below.

Cortical structures location profile (87 parameters):

The exhaustive list of cortical structures supported in GSI-RADS are as follows, for each atlas:

1. MNI: frontal, temporal, parietal, occipital, cerebellum, brain stem, lateral ventricle, 3rd ventricle, 4th ventricle, caudate, putamen, thalamus, subthalamic nucleus, globus pallidus, fornix, and extracerebral cerebrospinal fluid.
2. Harvard-Oxford: frontal pole, insular cortex, superior frontal gyrus, middle frontal gyrus, inferior frontal gyrus (pars triangularis, pars opercularis), precentral gyrus, temporal pole, superior temporal gyrus (anterior and posterior division), middle temporal gyrus (anterior and posterior division), inferior temporal gyrus (anterior, posterior, and temporooccipital division), postcentral gyrus, superior parietal lobule, supramarginal gyrus (anterior and posterior division), angular gyrus, lateral occipital cortex (superior and inferior division), intracalcarine cortex, frontal medial cortex, juxtapositional lobule cortex, subcallosal cortex, paracingulate gyrus, cingulate gyrus (anterior and posterior divison), precuneous cortex, cuneal cortex, frontal orbital cor-

tex, parahippocampal gyrus (anterior and posterior divison), lingual gyrus, temporal fusiform cortex (anterior and posterior division), temporal occipital fusiform cortex, occipital fusiform gyrus, frontal operculum cortex, central operculum cortex, parietal operculum cortex, planum polare, heschl gyrus (including H1 and H2), planum temporale, supracalcarine cortex, occipital pole.

3. Schaefer7: visual, somatomotor, dorsal attention, salience ventral attention, limbic, front parietal control, and default.
4. Schaefer17: visual central, visual peripheral, somatomotor A, somatomotor B auditory, dorsal attention A, dorsal attention B, salience ventral attention A, salience ventral attention B, limbic orbito-frontal, limbic temporopolar, front-parietal control A, front-parietal control B, front-parietal control C, default A, default B, default C, and temporoparietal.

Subcortical structures location profile (2 × 68 parameters):

The exhaustive list of subcortical structures supported in GSI-RADS are as follows, for each atlas:

1. BCB: anterior commissure, anterior thalamic projections (left/right), arcuate anterior segment (left/right), arcuate long segment (left/right), arcuate posterior segment (left/right), cingulum (left/right), cingulum anterior (left/right), cingulum posterior (left/right), corpus callosum, cortico spinal (left/right), face U tract (left/right), fornix, frontal aslant tract (left/right), frontal commissure (left/right), frontal inferior longitudinal (left/right), frontal orbito polar (left/right), frontal superior longitudinal (left/right), fronto insular tract 1/2/3/4/5 (left/right), fronto marginal tract (left/right), fronto striatal (left/right), handinf U tract (left/right), handmid U tract (left/right), handsup U tract (left/right), inferior fronto occipital fasciculus (left/right), inferior longitudinal (left/right), optic radiations (left/right), paracentral U tract (left/right), pons (left/right), superior longitudinal fasciculus I/II/III (left/right), uncinate (left/right).

3. Metrics and measurements

For further taking into account multifocal tumors, instance-wise metrics were extended to consider both patient-wise and object-wise configurations.

Instance-wise:

In addition to the instance-wise performances (i.e., recall, precision, and F1-score) reported at a patient level (i.e., in a patient-wise fashion (PW)), an additional computation at the object level is proposed (i.e., in an object-wise fashion (OW)). In case of multifocal tumors, each foci was considered as a separate object for the object-wise computation.

4. Results

4.1. Architecture comparison

Performance comparison with the extended set of metrics is reported first hospital-wise in Table S2, and overall in Table S3.

Table S2: Hospital-wise segmentation and detection performances comparison between the two main architectures. For each hospital, the upper row represents nnU-Net and the lower row represents AGU-Net.

Hospital	Pixel-wise			Patient-wise (PW)				Object-wise (OW)		
	Dice	Dice-TP	HD95	FPPP	F1	Recall	Precision	F1	Recall	Precision
TIL	90.37 ± 10.42	90.95 ± 07.53	09.33 ± 28.80	0.19	95.93	99.34	92.74	87.95	88.41	87.50
	87.55 ± 13.74	89.22 ± 07.00	04.63 ± 06.51	0.02	98.35	98.03	98.67	89.06	81.73	97.83
HAG	89.23 ± 11.65	89.89 ± 06.62	22.59 ± 45.08	0.76	84.39	99.02	73.52	73.63	89.85	62.38
	84.68 ± 18.38	88.10 ± 07.07	04.57 ± 09.82	0.01	97.61	96.11	99.15	93.61	89.39	98.24
MIL	90.95 ± 06.60	90.95 ± 06.60	03.89 ± 11.70	0.09	98.13	100.0	96.33	93.11	92.66	93.57
	85.10 ± 17.24	87.42 ± 10.17	04.31 ± 07.76	0.02	97.98	97.33	98.64	93.18	88.88	97.91
ZWO	89.09 ± 11.97	90.28 ± 06.57	14.57 ± 32.33	0.33	92.39	98.61	86.91	82.55	86.73	78.76
	82.26 ± 18.28	84.61 ± 12.04	06.19 ± 08.75	0.09	96.46	97.22	95.71	89.55	87.36	91.86
VIE	91.70 ± 03.73	91.70 ± 03.73	05.68 ± 16.43	0.12	97.35	100.0	94.83	90.84	89.31	92.42
	84.56 ± 15.97	86.64 ± 08.97	06.00 ± 09.92	0.02	98.17	97.59	98.76	85.41	75.78	97.84
ALK	87.98 ± 10.62	87.98 ± 10.62	24.95 ± 43.38	0.84	86.55	100.0	76.30	66.32	70.83	62.35
	81.32 ± 25.22	88.29 ± 08.69	03.22 ± 03.12	0.05	95.00	92.10	98.09	84.79	76.27	95.45
PAR	88.91 ± 12.14	90.13 ± 06.30	04.56 ± 11.81	0.06	97.62	98.64	96.62	97.10	100.0	94.38
	80.61 ± 22.08	86.11 ± 08.37	04.43 ± 04.15	0.01	96.16	93.24	99.28	97.46	96.25	98.70
SLO	88.21 ± 11.61	89.56 ± 06.98	05.30 ± 11.81	0.20	93.88	97.95	90.13	79.67	73.86	86.48
	82.54 ± 19.69	86.05 ± 10.11	10.93 ± 25.20	0.10	95.65	95.91	95.39	78.52	68.60	91.80
STO	82.14 ± 20.19	85.75 ± 11.93	09.73 ± 24.63	0.15	94.39	95.38	93.43	88.16	86.88	89.48
	77.78 ± 26.29	85.29 ± 12.22	06.35 ± 09.83	0.05	93.83	90.68	97.20	87.22	80.42	95.27
SFR	87.25 ± 13.47	88.57 ± 08.19	06.71 ± 20.78	0.09	97.48	98.50	96.49	86.25	80.27	93.19
	82.84 ± 17.28	84.73 ± 12.04	05.70 ± 08.39	0.07	97.36	97.76	96.96	81.91	72.81	93.63
GRO	87.15 ± 11.61	87.15 ± 11.61	30.27 ± 50.14	0.93	84.42	100.0	73.04	72.38	94.21	58.76
	85.46 ± 13.95	86.37 ± 11.18	08.45 ± 16.69	0.18	95.42	98.83	92.24	88.61	90.08	87.20
UTR	87.40 ± 10.87	88.36 ± 06.39	04.95 ± 12.57	0.14	96.62	98.83	94.50	90.47	90.94	90.00
	82.60 ± 13.82	84.03 ± 08.81	06.62 ± 09.49	0.10	97.13	98.24	96.05	89.28	86.83	91.89
AMS	87.93 ± 09.93	87.93 ± 09.93	09.73 ± 26.30	0.34	91.82	100.0	84.87	80.11	80.86	79.37
	83.39 ± 17.22	85.82 ± 10.70	07.56 ± 15.36	0	98.42	96.90	100.0	80.74	67.70	100.0

Table S3: Segmentation performances obtained with the two considered architectures using the P1 preprocessing scheme, averaged over the 13 folds of the GS1 subset. For each category, the upper row corresponds to nnU-Net and the lower row to AGU-Net. The tumor volume threshold for the small and large categories was set to 3 ml.

Category	Pixel-wise			Patient-wise (PW)				Object-wise (OW)		
	Dice	Dice-TP	HD95	FPPP	F1	Recall	Precision	F1	Recall	Precision
All	**86.88 ± 14.49**	**88.38 ± 01.65**	10.59 ± 27.94	0.26 ± 0.29	93.88 ± 04.81	**98.11 ± 01.23**	90.37 ± 08.50	85.69 ± 08.64	**87.34 ± 07.83**	84.96 ± 12.46
	82.14 ± 20.28	86.04 ± 01.49	**06.09 ± 10.82**	**0.05 ± 0.04**	**96.27 ± 01.39**	95.27 ± 02.44	**97.37 ± 02.04**	**87.71 ± 05.18**	81.67 ± 08.61	**95.23 ± 03.57**
Unifocal	87.74 ± 13.77	89.13 ± 01.60	10.74 ± 28.77	0.26 ± 0.29	93.47 ± 05.34	98.27 ± 01.36	89.53 ± 09.22	93.47 ± 05.34	98.27 ± 01.36	89.53 ± 09.22
	83.69 ± 18.83	86.98 ± 01.52	04.98 ± 09.41	0.04 ± 0.04	96.87 ± 01.38	96.03 ± 01.95	97.77 ± 01.95	96.87 ± 01.38	96.03 ± 01.95	97.77 ± 01.95
Multifocal	84.12 ± 16.08	85.94 ± 02.16	10.10 ± 24.69	0.25 ± 0.33	95.12 ± 04.66	97.60 ± 03.36	93.09 ± 07.92	83.32 ± 08.75	77.96 ± 11.29	90.66 ± 10.57
	77.15 ± 23.40	82.86 ± 03.34	09.66 ± 13.69	0.09 ± 0.09	94.26 ± 03.05	92.80 ± 06.68	96.10 ± 03.37	76.93 ± 08.99	65.56 ± 12.32	94.64 ± 04.85
Small	63.44 ± 28.01	73.66 ± 08.73	31.56 ± 43.84	0.53 ± 0.66	81.92 ± 11.59	84.74 ± 10.10	81.67 ± 16.66	77.89 ± 14.66	84.12 ± 11.28	74.19 ± 18.56
	45.21 ± 34.75	71.92 ± 08.18	09.11 ± 16.15	0.09 ± 0.11	72.06 ± 15.09	61.01 ± 20.55	93.62 ± 09.15	80.53 ± 17.34	74.57 ± 22.07	89.76 ± 11.69
Large	88.76 ± 10.60	89.41 ± 01.45	08.95 ± 25.34	0.24 ± 0.27	94.77 ± 04.56	99.18 ± 00.78	91.04 ± 08.03	86.41 ± 08.49	87.66 ± 08.09	86.02 ± 12.12
	85.11 ± 14.89	86.75 ± 01.75	05.84 ± 10.14	0.05 ± 0.05	97.79 ± 01.32	98.02 ± 01.22	97.58 ± 02.21	88.23 ± 05.01	82.33 ± 08.56	95.57 ± 03.78

4.2. External validity

External validity performances considering the whole range of metrics are reported in Table S4.

Table S4: Segmentation performances obtained over the GS2 subset with the two considered architectures and a baseline. The results are averaged from the 3 models trained following the custom validation protocol with P1 preprocessing.

Arch.	Pixel-wise			Patient-wise (PW)				Object-wise (OW)		
	Dice	Dice-TP	HD95	FPPP	F1	Recall	Precision	F1	Recall	Precision
H^2NF-Net	85.46	-	4.18	-	-	-	-	-	-	-
nnU-Net	84.07 ± 13.76	85.44 ± 0.22	4.07 ± 4.84	0.06 ± 0.01	97.73 ± 0.29	98.29 ± 0.27	97.19 ± 0.41	92.49 ± 0.27	89.89 ± 0.24	95.24 ± 0.80
AGU-Net	81.47 ± 23.31	87.27 ± 0.21	5.67 ± 8.60	0.11 ± 0.06	93.95 ± 0.90	93.17 ± 0.96	94.83 ± 2.84	86.26 ± 1.06	82.33 ± 1.91	90.85 ± 4.80

4.3. Preprocessing impact

Performance comparison with the extended set of metrics is reported in Table S5.

Table S5: Segmentation performances obtained with the different architectures and preprocessing schemes, averaged from the three models of the custom validation protocol, over the two data subsets.

Arch.	Subset.	Preproc.	Pixel-wise			Patient-wise (PW)				Object-wise (OW)		
			Dice	Dice-TP	HD95	FPPP	F1	Recall	Precision	F1	Recall	Precision
nnU-Net	GS1	P1	88.66 ± 11.52	89.36 ± 1.30	08.52 ± 25.58	0.19 ± 0.09	95.43 ± 2.39	99.21 ± 0.61	92.06 ± 4.38	85.37 ± 3.36	83.66 ± 3.70	87.43 ± 5.67
		P2	**89.14 ± 11.04**	**89.82 ± 1.14**	03.90 ± 10.23	0.06 ± 0.02	98.73 ± 0.56	**99.21 ± 0.61**	98.25 ± 0.55	**89.55 ± 2.34**	**84.35 ± 4.48**	95.62 ± 1.93
AGU-Net	GS1	P1	85.08 ± 15.48	86.80 ± 1.91	05.76 ± 10.09	0.03 ± 0.03	98.15 ± 0.31	97.91 ± 0.66	98.41 ± 1.24	84.07 ± 3.50	74.44 ± 5.51	96.92 ± 2.64
		P2	87.42 ± 13.91	88.70 ± 1.19	**03.86 ± 6.70**	**0.02 ± 0.01**	98.72 ± 0.57	98.43 ± 0.70	**99.01 ± 0.76**	89.24 ± 2.95	81.90 ± 4.70	**98.21 ± 1.43**
nnU-Net	GS2	P1	84.38 ± 15.98	86.74 ± 1.08	18.11 ± 30.04	0.44 ± 0.13	89.90 ± 2.29	97.15 ± 0.16	83.74 ± 3.93	81.17 ± 3.48	90.93 ± 0.74	73.53 ± 5.91
		P2	84.07 ± 13.76	85.44 ± 0.22	04.07 ± 4.84	0.06 ± 0.01	97.73 ± 0.29	98.29 ± 0.27	97.19 ± 0.41	92.49 ± 0.27	89.89 ± 0.24	95.24 ± 0.80
AGU-Net	GS2	P1	81.47 ± 23.31	87.27 ± 0.21	05.67 ± 8.60	0.11 ± 0.06	93.95 ± 0.90	93.17 ± 0.96	94.83 ± 2.84	86.26 ± 1.06	82.33 ± 1.91	90.85 ± 4.80
		P2	87.04 ± 16.10	89.38 ± 0.46	04.44 ± 8.06	0.14 ± 0.05	95.56 ± 0.98	97.38 ± 1.60	93.84 ± 2.03	89.28 ± 1.63	89.35 ± 0.46	89.30 ± 3.62

Performance comparison with the extended set of metrics and model configurations is reported in Table S6. The use of a more complex preprocessing scheme (P3) with the AGU-Net architecture design slightly worsens overall performances. Best performances with this design is still achieved using the second preprocessing scheme (P2). Using a wider architecture, hence a higher number of parameters, leads to a small metrics increase across the board. The overall best performances with the AGU-Net design are obtained with the wider architecture and second preprocessing scheme. The best nnU-Net design still reaches a higher average Dice score by 1.2%, for slightly worse 95th percentile Hausdorff distance and F1-score, considering only the three folds of the custom validation protocol.

Table S6: Segmentation performances obtained with the different architectures and preprocessing schemes, averaged from the 3 models of the custom validation protocol, on the specified data subset.

Arch.	Subset.	Preproc.	Pixel-wise			Patient-wise (PW)				Object-wise (OW)		
			Dice	Dice-TP	HD95	FPPP	F1	Recall	Precision	F1	Recall	Precision
nnU-Net	P1	GS1	88.66 ± 11.52	89.36 ± 1.30	8.52 ± 25.58	0.19 ± 0.09	95.43 ± 2.39	99.21 ± 0.61	92.06 ± 4.38	85.37 ± 3.36	83.66 ± 3.70	87.43 ± 5.67
	P2	GS1	89.14 ± 11.04	89.82 ± 1.14	3.90 ± 10.23	0.06 ± 0.02	98.73 ± 0.56	99.21 ± 0.61	98.25 ± 0.55	89.55 ± 2.34	84.35 ± 4.48	95.62 ± 1.93
AGU-Net	P1	GS1	85.08 ± 15.48	86.80 ± 1.91	5.76 ± 10.09	0.03 ± 0.03	98.15 ± 0.31	97.91 ± 0.66	98.41 ± 1.24	84.07 ± 3.50	74.44 ± 5.51	96.92 ± 2.64
	P2	GS1	87.42 ± 13.91	88.70 ± 1.19	3.86 ± 6.70	0.02 ± 0.01	98.72 ± 0.57	98.43 ± 0.70	99.01 ± 0.76	89.24 ± 2.95	81.90 ± 4.70	98.21 ± 1.43
	P3	GS1	86.56 ± 15.04	88.19 ± 0.78	4.64 ± 8.28	0.03 ± 0.00	98.08 ± 0.76	97.91 ± 1.25	98.26 ± 0.28	87.89 ± 3.04	80.46 ± 4.81	96.99 ± 0.50
AGU-Net-W	P1	GS1	86.44 ± 15.63	88.39 ± 1.32	4.65 ± 7.92	0.01 ± 0.01	98.44 ± 0.13	97.65 ± 0.84	99.25 ± 0.61	86.36 ± 3.57	77.04 ± 5.46	98.53 ± 1.15
	P2	GS1	87.93 ± 13.98	89.15 ± 0.89	3.77 ± 6.67	0.01 ± 0.00	98.85 ± 0.28	98.43 ± 0.70	99.27 ± 0.24	89.64 ± 2.23	82.25 ± 3.96	98.64 ± 0.56
AGU-Net	P1	GS2	81.47 ± 23.31	87.27 ± 0.21	5.67 ± 8.60	0.11 ± 0.06	93.95 ± 0.90	93.17 ± 0.96	94.83 ± 2.84	86.26 ± 1.06	82.33 ± 1.91	90.85 ± 4.80
	P2	GS2	87.04 ± 16.10	89.38 ± 0.46	4.44 ± 8.06	0.14 ± 0.05	95.56 ± 0.98	97.38 ± 1.60	93.84 ± 2.03	89.28 ± 1.63	89.35 ± 0.46	89.30 ± 3.62
	P3	GS2	87.50 ± 15.85	89.80 ± 0.57	4.06 ± 6.48	0.26 ± 0.13	94.29 ± 1.82	97.38 ± 1.60	91.44 ± 3.30	85.87 ± 3.84	89.80 ± 0.45	82.61 ± 7.36
nnU-Net	P1	GS2	84.07 ± 13.76	85.44 ± 0.22	4.07 ± 4.84	0.06 ± 0.01	97.73 ± 0.29	98.29 ± 0.27	97.19 ± 0.41	92.49 ± 0.27	89.89 ± 0.24	95.24 ± 0.80
	P2	GS2	84.38 ± 15.98	86.74 ± 1.08	18.11 ± 30.04	0.44 ± 0.13	89.90 ± 2.29	97.15 ± 0.16	83.74 ± 3.93	81.17 ± 3.48	90.93 ± 0.74	73.53 ± 5.91
